# Statistically significant features improve binary and multiple Motor Imagery task predictions from EEGs

**DOI:** 10.3389/fnhum.2023.1223307

**Published:** 2023-07-11

**Authors:** Murside Degirmenci, Yilmaz Kemal Yuce, Matjaž Perc, Yalcin Isler

**Affiliations:** ^1^Department of Biomedical Technologies, Izmir Katip Celebi University, İzmir, Türkiye; ^2^Department of Computer Engineering, Alanya Alaaddin Keykubat University, Antalya, Türkiye; ^3^Faculty of Natural Sciences and Mathematics, University of Maribor, Maribor, Slovenia; ^4^Department of Medical Research, China Medical University Hospital, China Medical University, Taichung, Taiwan; ^5^Alma Mater Europaea, Maribor, Slovenia; ^6^Complexity Science Hub Vienna, Vienna, Austria; ^7^Department of Physics, Kyung Hee University, Seoul, Republic of Korea; ^8^Department of Biomedical Engineering, Izmir Katip Celebi University, İzmir, Türkiye

**Keywords:** brain-computer interfaces (BCIs), electroencephalogram (EEG), feature selection, machine learning, Motor Imagery (MI) task classification

## Abstract

In recent studies, in the field of Brain-Computer Interface (BCI), researchers have focused on Motor Imagery tasks. Motor Imagery-based electroencephalogram (EEG) signals provide the interaction and communication between the paralyzed patients and the outside world for moving and controlling external devices such as wheelchair and moving cursors. However, current approaches in the Motor Imagery-BCI system design require effective feature extraction methods and classification algorithms to acquire discriminative features from EEG signals due to the non-linear and non-stationary structure of EEG signals. This study investigates the effect of statistical significance-based feature selection on binary and multi-class Motor Imagery EEG signal classifications. In the feature extraction process performed 24 different time-domain features, 15 different frequency-domain features which are energy, variance, and entropy of Fourier transform within five EEG frequency subbands, 15 different time-frequency domain features which are energy, variance, and entropy of Wavelet transform based on five EEG frequency subbands, and 4 different Poincare plot-based non-linear parameters are extracted from each EEG channel. A total of 1,364 Motor Imagery EEG features are supplied from 22 channel EEG signals for each input EEG data. In the statistical significance-based feature selection process, the best one among all possible combinations of these features is tried to be determined using the independent *t*-test and one-way analysis of variance (ANOVA) test on binary and multi-class Motor Imagery EEG signal classifications, respectively. The whole extracted feature set and the feature set that contain statistically significant features only are classified in this study. We implemented 6 and 7 different classifiers in multi-class and binary (two-class) classification tasks, respectively. The classification process is evaluated using the five-fold cross-validation method, and each classification algorithm is tested 10 times. These repeated tests provide to check the repeatability of the results. The maximum of 61.86 and 47.36% for the two-class and four-class scenarios, respectively, are obtained with Ensemble Subspace Discriminant among all these classifiers using selected features including only statistically significant features. The results reveal that the introduced statistical significance-based feature selection approach improves the classifier performances by achieving higher classifier performances with fewer relevant components in Motor Imagery task classification. In conclusion, the main contribution of the presented study is two-fold evaluation of non-linear parameters as an alternative to the commonly used features and the prediction of multiple Motor Imagery tasks using statistically significant features.

## 1. Introduction

Brain–computer Interfaces (BCIs) help to establish and realize the interaction between humans and computers using physiological signals acquired from the brain (Tiwari et al., 2022). It allows individuals who can not control a part of their resulting from paralysis or similar diseases but who are conscious to communicate with the outside world and control the robot arm, wheelchair, computer, and similar devices with thought power. The basic concept of BCIs is based on capturing brain's electrical signals, analyzing them on the artificial intelligence-powered software, and converting them to emotions and thoughts for particular purposes. The first step of BCI system design is data acquisition to obtain physiological signals. A neuron captures the information about any thought, which is passed to the other neurons after being processed. This communication among neurons generates electrical activities that can be measured from the body surface (Tan and Nijholt, 2010; Bansal and Mahajan, 2019). If these activities are originated from the brain, they can be captured using electroencephalography (EEG) visualization devices. EEG is a non-invasive method of placing electrodes over the scalp (just as near to the brain cortex as possible) (Yuan and He, 2014; Tiwari et al., 2022). Motor Imagery (MI) EEG signals are acquired during mental tasks. In the field of BCI, MI signals are generated when the subject only imagines a movement of a body part without actually performing it (Musallam et al., 2021). Similar to other BCI systems, MI-based BCI systems' goal is to control one or more extrinsic devices by translating EEG signals into commands (Tiwari et al., 2022). Hence, the processing of these signals plays an important role in the design of assistive devices for motor-disabled and paralyzed persons (Degirmenci et al., 2022c).

In recent studies, the traditional handcrafted feature extraction processes have been studied to classify MI tasks. These studies analyze EEG signals using traditional machine learning methods. The handcrafted feature extraction process includes some basic and definite steps after acquiring EEG signals from subjects. These are signal preprocessing, feature extraction, feature selection, and classification. Among these steps, the feature extraction and selection processes play an important role in EEG-based studies (Degirmenci et al., 2022b). The preprocessing step includes different and significant operations such as signal filtering, signal normalization, artifact removal, and signal segmentation (Altaheri et al., 2021). In the feature extraction step, various approaches have been introduced to extract task-related intrinsic information from EEG signals by researchers. The MI features are separated into three categories based on the processing domain which are temporal features, spectral features, and spatial features.

Temporal features are supplied from the time domain of signals using time points or different time segments and include features such as mean value, kurtosis, variance, skewness, root mean square value, and Hjorth parameters (Pawar and Dhage, 2020; Degirmenci et al., 2022b). Spectral features contain both frequency domain features such as power spectral density and fast Fourier transform (Djamal et al., 2017; Degirmenci et al., 2022c) and time-frequency domain features such as short-time Fourier transform (Ha and Jeong, 2019) and Wavelet transform (Chaudhary et al., 2020). Spatial features supply information about particular electrode locations on the brain cortex. The common spatial patterns (Blanco-Diaz et al., 2022) and its different versions such as sparse common spatial patterns (Arvaneh et al., 2011), stationary common spatial patterns (Samek et al., 2012), divergence common spatial patterns (Samek et al., 2013), probabilistic common spatial patterns (Wu et al., 2014), and filter bank common spatial patterns (Ang et al., 2012) are the mostly-studied feature extraction methods to capture spatial information in MI task classification. The compatibility of all these different feature categories with the non-stationary structure of the EEG is important in determining the features to be used. According to the non-stationary structure of the EEG signals, the spectral components of the these signals change as a function of time. Thus, signal processing the EEG signals only in the time-domain or the frequency-domain might not be sufficient to provide information about the spectral characteristics of the EEG signals (Boashash, 2015). The combination of these different categories should be analyzed in accordance with the nature of the EEG signals, using time-frequency domain features and non-linear parameters, in addition to the frequently used time-domain, frequency-domain, and spatial-domain features.

Although there are different MI task features to analyze within MI EEG signals, the correlations among these features are significant for algorithm performance. The simultaneous combination of a large number of various features unnecessarily increases the complexity of classifiers due to confusion caused by redundant information in the feature set. In addition, the classifier performance decreases in some cases due to this confusion (Hart et al., 2000; Isler, 2009; Narin et al., 2014). As one of the solutions to this problem, all possible feature subsets can be defined and the separability of each feature subset can be evaluated based on classifier performance. Then, the relevant feature subset which provides the highest separability between MI tasks can be determined. Unfortunately, when too many features are studied, too many combinations need to be tried to explore the relevant and effective feature set. However, such an approach requires and results in the computational load of classifier algorithms (Narin et al., 2014). These feature selection algorithms are classified into two main groups based on whether they consider a certain criterion for classifier performance or not, which are wrapper and filter approaches, respectively (Blum and Langley, 1997; Kohavi and John, 1997; Guyon and Elisseeff, 2003). Statistical significance-based selection, backward elimination, forward selection, principal component analysis (PCA), and genetic algorithms (GAs) are mostly used feature selection algorithms to analyze biomedical signals in the literature (Isler, 2009; Narin et al., 2014; Mousa et al., 2016). In the classification process, various machine learning algorithms have been computed to classify MI tasks such as Naive Bayes, k-nearest neighbors (k-NN), linear discriminant analysis, support vector machine, multi-layer perceptron, radial basis function, extreme learning machine, and deep neural network (Meziani et al., 2019; Degirmenci et al., 2022b,c; Tiwari et al., 2022). This study aims to introduce an effective approach for MI task classification using various features of EEG signals and different machine learning algorithms. The main contributions of this study can be highlighted as follows:

- We investigated a multi-directional handcrafted feature extraction-based approach that makes use of different feature categories including temporal, spectral, and non-linear features.
- We implemented the non-linear feature extraction method computing Poincare plot measurements of EEG signals to ensure the information about non-linear dynamics of signals in MI task classification.
- We investigated the performance effect of the statistical significance-based feature selection method on MI task classification.
- We comparatively evaluated the performance effect of the seven different machine learning algorithms with the combination of different MI EEG features.

In the following section, we preferred to give a brief review of MI task studies separately from this introduction. Then, we gave methods and materials as a new section to explain the dataset used in addition to feature extraction, statistical significance-based feature selection, classifier algorithms, and performance evaluation metrics utilized in this study. Next, we introduced all the achieved classifier performances in the Results section. In the last two separate sections, we discussed what these results mean and we concluded the outcomes of the study, respectively.

## 2. Related works

The feature extraction and feature selection methods are critical steps for the prediction of MI-based EEG tasks since these steps have a direct impact on the classification performance. In the literature, different approaches were tested to extract MI features and determine which of them gives higher classifier performances than other feature and classifier combinations. MI EEG signals supply the temporal, spectral, and spatial features from their intrinsic structure. These features extracted different features from different categories can be combined to classify MI tasks in research studies. In 2022, Degirmenci et al. presented a temporal feature extraction-based approach that uses 24 different time-domain features. They also investigated the effectiveness of the statistical significance (ANOVA)-based feature selection process for the classification of the four MI tasks (Degirmenci et al., 2022b). In classification, 11 various machine learning algorithms were tested, and the maximum average accuracy value was found as 44.00% using linear discriminant analysis. In a study conducted by Hamedi et al., integrated EEG (IEEG) and root mean square (RMS) measures were extracted from the time domain of EEG signals. In the classification of the three-class MI task, the effectiveness of neural network-based algorithms, which are multi-layer perceptron and radial basis function neural networks, was investigated. The results revealed that RMS was more capable than IEEG for differentiating MI tasks, and radial basis function was more accurate and faster than multi-layer perceptron (Hamedi et al., 2014). The fast Fourier transform is one of the most applied methods to extract spectral features. In the study by Jusas and Samuvel (2019), band power, time domain parameters, fast Fourier transform, and channel variance were evaluated for the feature extraction process, and different feature selection methods, which are PCA, sequential forward selection, sequential backward selection, locality preserving projections, and local Fisher discriminant analysis, were investigated. They concluded that the combination of fast Fourier transform and covariance matrix-based feature extraction with PCA-based feature selection supplied the best classification performance among all combinations. In the literature, recent studies have investigated the effect of EEG sub-bands using fast Fourier transform-based frequency band extraction. In 2019, Isa et al. presented a binary MI task classification study based on the EEG frequency band extraction using the fast Fourier transform. The linear discriminant analysis was applied over these spectral features to minimize the number of feature dimensions. They evaluated the maximum accuracy value as 79.23% using the Naive Bayes algorithm for the classification of right-hand and left-hand tasks (Isa et al., 2019). The main drawbacks of fast Fourier transform are two-fold: it is non-suitable for the non-stationary characteristic of EEG signals and it does not include time information. Therefore, different methods which include time and frequency information were used to extract spectral features. Short-time Fourier transform is one of the time-frequency representation techniques that process the local characteristics of a signal utilizing a window. It supplies the spectral features using the time-frequency domain. In the study by Ha and Jeong (2019), the authors proposed a method for binary classification of MI tasks using the short-time Fourier transform, and a capsule network (CapsNet). EEG signals were converted to 2D images using the short-time Fourier transform and these images were classified with CapsNet and other well-known machine learning algorithms. They concluded that CapsNet-based classification outperforms all the other machine learning-based classifications with an average classification accuracy of 78.44% in their presented study. Other time-frequency domain features can be extracted using Wavelet Transform. It supplies multi-resolution analysis from EEG signals using several filters with different bandwidths (Ha and Jeong, 2019). In a study presented by Luo et al., the effect of the Wavelet packet decomposition-based EEG subband extraction approach was investigated for the binary classification (right-hand and left-hand movement tasks). They also applied the Dynamic frequency feature selection (DFFS) method to reduce the extracted features. They calculated the average accuracy value of 68.32% using random forest algorithm (Lu et al., 2020). In spatial feature extraction, the common spatial pattern algorithm is the most preferred method which uses spatial filters. In the study by Kato et al. (2020), a five-class MI task classification study based on the multi-class common spatial patterns method was proposed. Five different finger movements were differentiated with an accuracy of 40.60% using support vector machine. Unfortunately, the common spatial patterns' drawback is the manual frequency band selection based on individual structures. The filter-bank common spatial pattern method, which utilizes several different frequency bands in parallel, has been presented to overcome this problem. Adopting the FBSCP method improved the classification performance for MI task studies (Ha and Jeong, 2019). In 2008, a common spatial pattern and filter-bank common spatial pattern-based MI task classification study is performed using publicly available BCI competition III dataset IVa. In the Naive Bayesian Parzen Window (NBPW)-based classification, filter-bank common spatial pattern yielded superior averaged test accuracy of 81.10%, while the common spatial patterns-based approach yielded an accuracy of 73.30%. They concluded that filter-bank common spatial patterns supplied statistically outstanding performance than common spatial patterns (Ang et al., 2008).

In recent studies, it has been observed that three different feature categories are generally used in the feature extraction process for MI-based EEG signals, but it has been noted that the most studied feature extraction approaches are the spectral domain and spatial domain features. Unfortunately, the effect of non-linear features on MI task classification has not been studied much. The Poincare plot measurements are one of the non-linear feature extraction methods that were studied in the analysis of different biomedical signals and supplied high classification results. Its simple visual interpretation and its proven clinical ability as a predictor of disease and cardiac dysfunction made this technique popular in the analysis of different physiological signals (Isler and Kuntalp, 2007; Isler, 2009; Narin et al., 2014; Isler et al., 2019). Taking into account its performance in other studies (Isler and Kuntalp, 2009; Cancioglu et al., 2021), Poincare plot measurements can be an effective method for non-linear dynamics of EEG signals that complicate the processing of them. Considering the contributions and deficiencies of the existing studies, in this study, a feature extraction method based on the combination of Poincare plot measurements from the non-linear feature extraction methods with temporal features and spectral features is implemented for MI task classification.

## 3. Materials and methods

**Figure 1:**
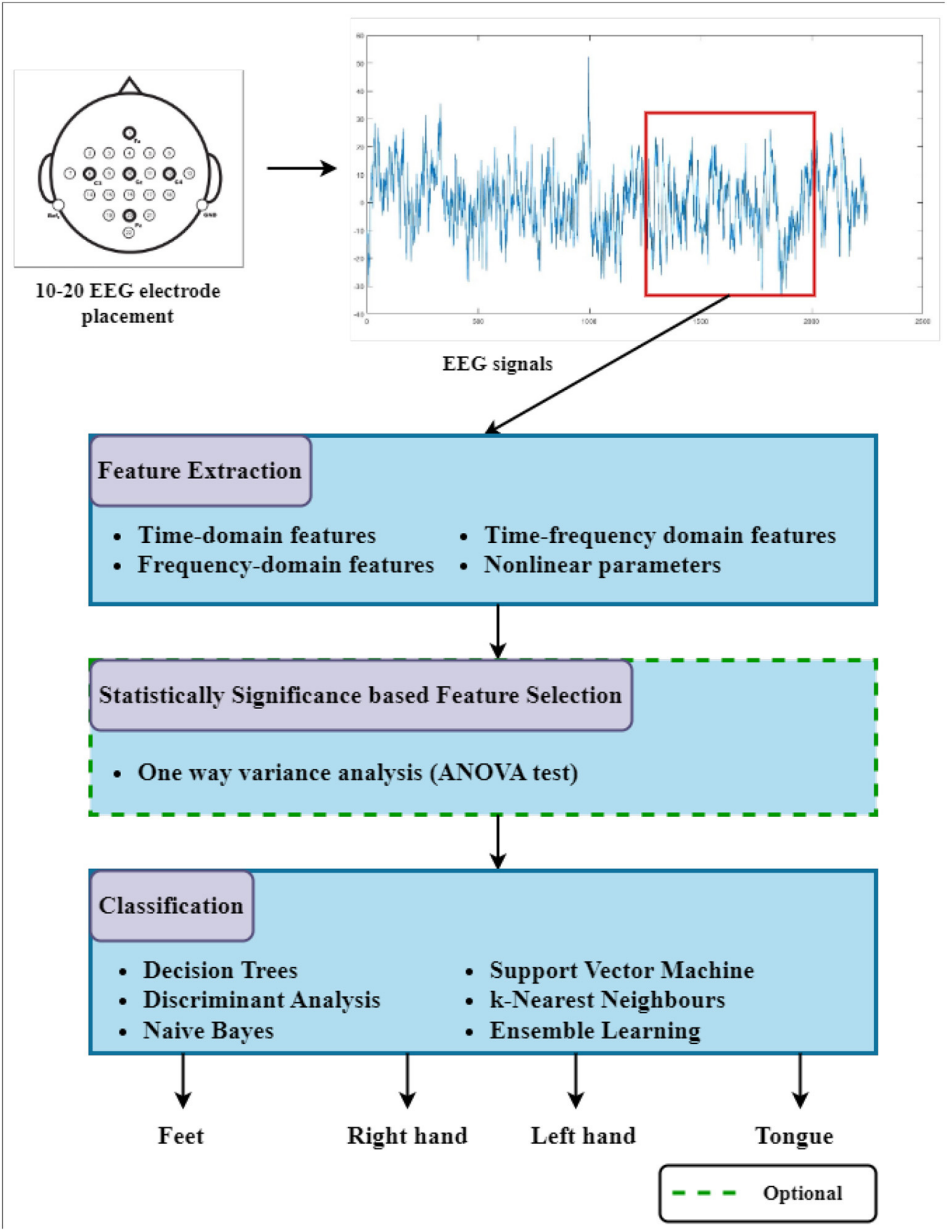
The block diagram of the suggested multi-class Motor Imagery task classification study. Three-second segments from EEG signals are used for the feature extraction process. Well-known classifiers are tested to discriminate the BCI command using selected features among extracted features (The dashed line representation refers to analyses in which ANOVA-based feature selection is applied, and statistically significant features are applied to classifiers instead of all features.).

**Figure 2:**
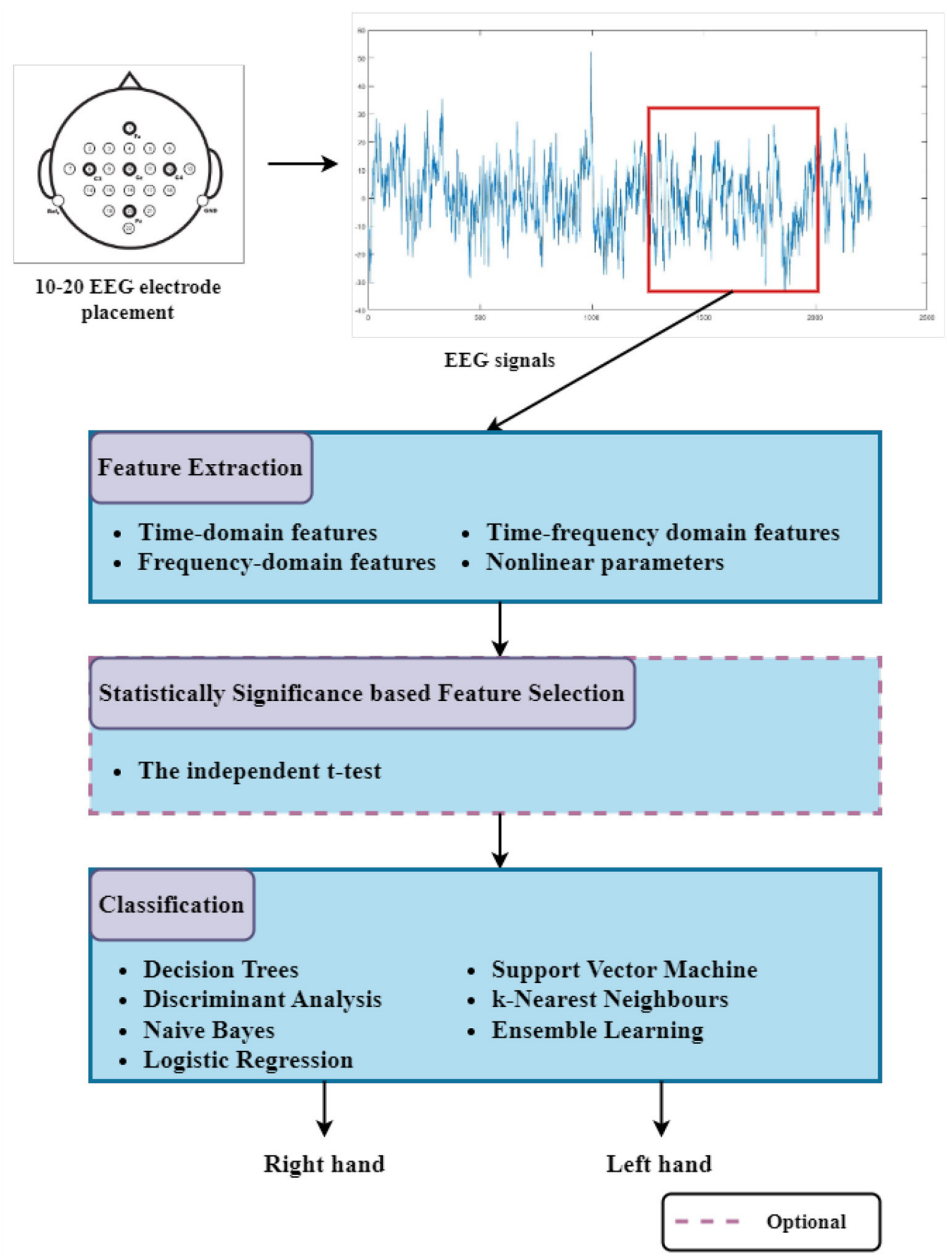
The block diagram of the suggested binary class Motor Imagery task classification study. Three-second segments from EEG signals are used for the feature extraction process. Well-known classifiers are tested to discriminate the BCI command using selected features among extracted features (The dashed line representation refers to analyses in which the independent *t*-test based feature selection is applied, and statistically significant features are applied to classifiers instead of all features.).

In this section, the EEG dataset and methodologies that are adopted and used for feature extraction, feature selection, and classification for MI-based EEG signals are described in detail. The flowcharts of the suggested multi-class and binary class Motor Imagery task classification studies are presented in Figures 1, 2, respectively.

### 3.1. Dataset

In this study, the publicly available BCI Competition IV Dataset IIa was used to evaluate the performance of the classifier methods for binary and multiple MI task classification (Brunner et al., 2008). The dataset contains the EEG and EOG signals, which were captured and recorded using 22 EEG channels and 3 EOG channels, respectively. EEG signals were recorded using 22 Ag/AgCl electrodes, and the sampling rate was defined as 250 Hz. The signals were collected for four different MI tasks which are the imagination of movement of the left hand (LH), right hand (RH), feet (F), and tongue (T) from 9 subjects of which 4 were females and 5 were males. Two sessions were organized to collect EEG signals on different days, and each session includes 6 runs separated by breaks. In each run, 48 different MI tasks were available, and these trials were designed to be 12 MI tasks for each of the four classes. During the recording, a visual cue was shown to the subject to imagine the movements for four different tasks. The preprocessing step of EEG signals includes a band-pass filtering process between 0.5 and 100 Hz and an additional 50 Hz notch filter application to eliminate line noise for this dataset.

### 3.2. Feature extraction

Initially, the relevant MI EEG segments, where EEG tasks were performed, are decomposed from original EEG signals for the feature extraction process. In this study, we extracted four feature sets of MI EEG features for the classification of MI task segments. The first set includes temporal features that are supplied from time-domain information of EEG segments. In the second set, spectral features are extracted using the fast Fourier transform-based frequency domain information of EEG segments. As a third set, time-frequency features are calculated based on Wavelet Transform. Finally, in the last set, Poincare plot measurements are calculated to extract non-linear features.

First, the relevant and distinctive temporal features are extracted based on the time-domain information of EEG signals. A total of 24 different temporal features, which include information about amplitude and statistical changes of the EEG signals, are supplied for each EEG segment (Sayilgan et al., 2021a; Degirmenci et al., 2022b). These temporal features are minimum, maximum, mean, standard deviation, integrated EEG value, mean absolute value, simple square integral value, variance, root mean square value, waveform length value, average amplitude change value, absolute difference in standard deviation, mode value of the signal, kurtosis, skewness, Hjorth parameters (activity, mobility, and complexity) inter-quarter intervals (1st quartile, 2nd quartile, and 3rd quartile), zero crossing, slope-change value, and signal range.

Next, the EEG subbands' energy, variance, and entropy values, and these values are calculated based on the frequency distribution of EEG signals. Hence, these spectral features include the information about frequency distribution embedded in EEG signals (Degirmenci et al., 2022c). The different oscillations are embedded in EEG signals which are known to be liable for various cognitive brain functions (Cura and Akan, 2021). These are known as delta (*δ*), theta (*θ*), alpha (*α*), beta (*β*), and gamma (γ) waves. The frequency bands of these waves are identified as delta (0.5–4 Hz), theta (4–8 Hz), alpha (8–13 Hz), beta (13–30 Hz), and gamma (30–100 Hz) for this study. The delta, theta, alpha, beta, and gamma bands are decomposed from the frequency distribution of MI EEG signals using the fast Fourier transform, and the energy, variance, and entropy values of these bands are calculated as spectral features. In the various EEG-based classification problems, machine learning-based approaches commonly use the energy, variance, and entropy values of EEG subbands, which are calculated from the frequency domain of signals as spectral features (Sayilgan et al., 2021c). Here, energy, variance, and entropy of frequency bands are calculated in the study by Sayilgan et al. (2021a) and Degirmenci et al. (2022c) as follows:


(1) $$\begin{array}{l} Energy_{f}=\overset{M}{\underset{i=1}{\sum }}y{\left(i\right)}^{2} \end{array}$$

(2) $$\begin{array}{l} Variance_{f}=\frac{1}{M-1}\cdot \overset{M}{\underset{i=1}{\sum }}{\left(y_{i}-\bar{y}\right)}^{2} \end{array}$$

(3) $$Entropy_{f}=\frac{1}{log(M)}\cdot \sum_{i=1}^{M}P(y(i))log(P(y(i))$$

Here, the energy of each frequency band is calculated based on the power spectrum, and f indicates the type of EEG subbands which are *δ*, *θ*, *α*, *β*, and *γ*. *Energy*_*f*_ corresponds to the energy of a frequency band, and M corresponds to the maximum frequency. The Fourier Transform of the EEG segment is indicated as *y*. *Variance*_*f*_ corresponds to the variance of a frequency band, and $\bar{y}$ denotes the average of the y signal. The spectral entropy measures the regularity of the power spectrum of the EEG signal, and *Entropy*_*f*_ corresponds to the entropy of a frequency band. *P*(*y*(*i*)) indicates the probability that the signal is in the given frequency domain.

Then, Wavelet Transform-based feature extraction process is conducted to calculate time-frequency features. EEG signals have non-stationarities and their spectral features do not include any time information. Wavelet Transform uses both time and frequency information and supplies multi-resolution analysis using several filters and bandwidths. It is a smooth and fast oscillation function that is well-localized in frequency and time (Sayilgan et al., 2021b). It can be used as a specially prepared dual Finite-Impulse Response (FIR) filter. The high-frequency and low-frequency components of EEG signals are extracted using frequency responses of FIR filters. Half of the data sampling rate is known as Nyquist frequency. The dividing point of the signal frequency is generally between 0 Hz and the specified Nyquist frequency. The same wavelet coefficients are employed in both low-pass (LP) and high-pass (HP) filters for the multi-resolution algorithm of Wavelet Transform (Gandhi et al., 2011). The LP filter coefficients are linked with a scaling parameter that defines the oscillatory frequency and the length of the wavelet, whereas the HP filter is linked with the wavelet function. The outputs of the LP filters and HP filters are denoted as the approximation *(a)* coefficients and detail *(d)* coefficients, respectively. EEG time signals can be completely divided into *(a)* and *(d)* coefficients depending on the decomposition level. The analysis of different statistical and non-statistical parameters over time and frequency can be performed by applying the Wavelet Transform to EEG signals. The subsets of the relevant coefficients of decomposition levels are categorized based on the frequency domain of EEG subbands for the extraction of EEG frequency bands. In this study, the Wavelet packet decomposition-based EEG subband extraction is used to calculate time-frequency features. The MI EEG signals are decomposed into seven decomposition levels. The approximation *a*_*i*_ and detail *d*_*i*_ coefficients were obtained for the decomposition levels of i = 1, 2,..., 7 for 250 Hz sampling frequency.

The various Discrete Wavelet Transform functions (Haar, Db2, Sym4, Coif1, Bior3.5, and Rbior2.8) can be used in Wavelet Transform-based feature extraction. There are several types of mother wavelets; therefore, determining a suitable mother wavelet is an important step. In the study by Sayilgan et al. (2021a), researchers conducted a study to define the effective wavelet function in steady-state visual-evoked potential (SSVEP) signals. The results of the study showed that the most successful wavelet function was the Haar wavelet. Hence, in this study, the Haar wavelet function was applied to the Wavelet packet decomposition process. MI EEG signals are subdivided into frequency bands (*δ*, *θ*, *α*, *β*, and *γ*) from *a*_*i*_ and *d*_*i*_ coefficients. The energy, variance, and entropy of these frequency bands are calculated as time-frequency features. The energy of each decomposition level was computed corresponding to the following equation (Gandhi et al., 2011):


(4) $$Energy_{d_{i}}=\sum_{j=1}^{N}|d_{i}{}_{j}{|}^{2},i=1,2,3,...,l$$

(5) $$Energy_{a_{i}}=\sum_{j=1}^{N}|a_{i}{}_{j}{|}^{2},i=1,2,3,...,l$$

In the equations, detail (*d*_*i*_) and approximate (*a*_*i*_) coefficients are used to supply subsets of each EEG frequency band (*δ*, *θ*, *α*, *β*, and *γ*) from the decomposition tree. The *(a)* and *(d)* coefficients of these frequency band subsets are denoted with *d*__*i*_*j*_ and *a*__*i*_*j*_, respectively. *i = 1,2,3,l* corresponds to the wavelet decomposition level that takes value from 1 to *l*. The number of *d* and *a* coefficients at each decomposition level is indicated with *N*.

By using the following equation, the entropy of each decomposition level is calculated (Isler, 2009).


(6) $$Entropy_{i}=\sum_{j=1}^{N}d_{i}{{}_{j}}^{2}log(d_{i}{{}_{j}}^{2}),i=1,2,3,...,l$$

The variance of each decomposition level is computed as follows (Gandhi et al., 2011):


(7) $$\begin{array}{l} Variance_{i}=\frac{1}{N-1}\cdot \overset{N}{\underset{j=1}{\sum }}{\left({d{}_{i}}_{j}-\mu_{i}\right)}^{2},i=1,2,3,...,l\\ \;\mu_{i}=\frac{1}{N}\cdot \overset{N}{\underset{j=1}{\sum }}{d{}_{i}}_{j},i=1,2,3,...,l \end{array}$$

Hence, μ_*i*_ expresses the mean of the decomposition level.

In the last feature extraction process, the non-linear parameters are extracted in addition to the temporal, spectral, and time-frequency features. MI EEG signals have non-linear dynamics in their characteristics. In recent studies, Poincare plot measures were commonly used as non-linear measures to analyze the different EEG signals. It characterized the non-linear dynamics inherent in the signal. The Poincare plot is a graph of each EEG sample (*x*_*i*_) on the x-axis and the next EEG sample (*x*_*i*+*lag*_) on the y-axis (Isler, 2009). In the x and y axes, (*x*_*i*_) and (*x*_*i*+*lag*_) intervals are placed to ensure the Poincare plot, respectively. The Poincare plot-based feature extraction process is adopted for this study, considering its favorable outcomes in the literature such as its simple visual interpretation and proven clinical ability (Isler and Kuntalp, 2007; Isler, 2009; Narin et al., 2014; Isler et al., 2019; Cancioglu et al., 2021). These drawings are procured from raw MI EEG segment data after defining (*x*_*i*_) and (*x*_*i*+*lag*_) intervals within EEG segments. An ellipse is fitted to the Poincare plot graph, and the standard deviation of the distance of the points on these plots indicates the width (*SD*1) and length (*SD*2) of the ellipse (Brennan et al., 2001). Poincare plot measures can be calculated as follows (Isler, 2009; Isler and Kuntalp, 2009):


(8) $$\begin{array}{l} x_{i}=\left(x_{0},x_{1},...,X_{N-m}\right) \end{array}$$

(9) $$\begin{array}{l} x_{i+lag}=\left(x_{m},x_{m+1},...,X_{N}\right) \end{array}$$

(10) $$\begin{array}{l} x_{a}=\frac{x_{i+lag}-x_{i}}{\sqrt{2}}\\ x_{b}=\frac{x_{i+lag}+x_{i}}{\sqrt{2}} \end{array}$$

(11) $$\begin{array}{l} SD_{1}=SD\left(x_{a}\right)\\ SD_{2}=SD\left(x_{b}\right) \end{array}$$

where *x*_*i*_ and *x*_*i*+*lag*_ represent the EEG segment data and the next EEG data interval in the Equations (8) and (9), respectively. With respect to defined intervals, *SD*_1_ and *SD*_2_ measurements were calculated utilizing Equations (10) and (11). *SD* indicates the standard deviation of the extracted time interval vectors in Equation (11). The *m*-lagged Poincare plot measurements were conducted to define different intervals. *SD*_1_ and *SD*_2_ measurements are calculated considering *lag=m* and m was set as 1 and 9 for this study. In this study, Poincare plot measures for *lag=9* were also calculated due to the positive effect on MI EEG signal classification (Degirmenci et al., 2022a). In our previous study (Degirmenci et al., 2022a), we investigated the performances of different feature vectors which were extracted from 10 lag values and the feature vector which is the combination of these vectors, separately. The results demonstrated that the most discriminative and effective feature set is the ninth feature vector that includes the features extracted when the lag value is defined as 9. The values of (*SD*1) and (*SD*2), for which we determined the m values as 1 and 9, were calculated. In addition, in addition to (*SD*1) and (*SD*2) calculations, the products (*SD*_1_*SD*_2_) and the rates (*SD*_1_/*SD*_2_) are calculated to investigate the relationships between these components. A total of four non-linear features were extracted for *lag=1* condition. In our Poincare plot process, eight non-linear features were extracted from *lag=1* and *lag=9* conditions for each EEG segment.

### 3.3. Statistical significance-based feature selection

The feature selection process aims to determine the relevant and effective features that will supply the highest discrimination between the classes of interest and also can minimize the complexity of classifiers (Isler et al., 2023). In this study, the statistical significance-based feature selection is applied to indicate the most effective combination of temporal, spectral, time-frequency, and non-linear features which provides the best discrimination of the MI tasks (Narin et al., 2014; Sayilgan et al., 2021b; Degirmenci et al., 2022b). This statistical significance-based feature selection approach is applied for each MI EEG feature set separately. In this study, two different classification models, which are binary and multi-class MI task classifications, are studied. Hence, two different types of statistical significance-based feature selection were used, i.e., the independent *t*-test and one-way analysis of variance (ANOVA test). The selected tests were determined considering the class number of the classification models. In binary classification, the independent *t*-test, which is commonly applied to define the significance of differences between measures of two different classes, is used for feature selection (Narin et al., 2014; Degirmenci et al., 2022c). In multi-class classification, the ANOVA test is adopted for feature selection (Bulut et al., 2022; Degirmenci et al., 2022b). ANOVA is a test applied when it is required to determine whether there is a difference between the means in conditions where there are two or more groups. Thus, the effects of the independent *t*-test and ANOVA test-based feature selection methods were investigated with temporal, spectral, time-frequency, and non-linear features. The statistical significance of every MI EEG feature were defined by calculating *p*-values. The statistical significances are measured based on the statistical significance level (α) equal to 0.05. A total of two feature sets containing the features that provide the statistical evidence range were obtained after the significant features were determined using the feature selection models (the independent *t*-test and ANOVA) for both two classification models. These selected feature vectors were given to the classification algorithms as input data to predict the MI tasks.

### 3.4. Classification

In this study, the MI EEG features described in the previous feature extraction section are used to predict MI tasks of EEG segments. We also compare the performance of the binary (RH and LH) and multi-task (RH, LH, F, and T) classifications using extracted features from temporal, spectral, time-frequency, and non-linear methods. The different versions of six different basic classifiers are computed to classify the extracted features (Hart et al., 2000). Hence, 24 different classification methods are tested considering the different sub-parameters of 6 classifier algorithms (Sayilgan et al., 2021a; Degirmenci et al., 2022a). The set of classifiers contains decision trees (fine, medium, and coarse), discriminant analysis (linear, quadratic), Naive Bayes (Gaussian, Kernel), support vector machine (linear, quadratic, cubic, fine Gaussian, medium Gaussian, and coarse Gaussian), k-NN (fine, medium, coarse, cubic, cosine, and weighted), and ensemble learning (boosted, bagged, subspace discriminant, subspace k-NN, and RUSBoosted Trees) algorithms. All these classification algorithms with different sub-parameters are available in the “Classification Learner” application of Matlab. Additionally, the logistic regression algorithm is tested for binary classification (Degirmenci et al., 2022c).

#### 3.4.1. Decisions trees

The decision tree is a machine learning algorithm that can divide the data into several different sub-groups and can also be utilized for classification outside of the regression process. The characteristic tree-like structure of this algorithm which includes branches and nodes gives the name of the algorithm (Tzallas et al., 2009). The training process is carried out based on learning a set of decision rules. A leaf node is created when the decision is made, whereas a decision node which is another branch is generated when the decision is not definite (Cura and Akan, 2021). In the decision tree-based classification process, the fine, medium, and coarse algorithms are used for this study.

#### 3.4.2. Discriminant analysis

The discriminant analysis classifier is one of the pattern recognition methods, and its main purpose is to correctly divide the independent variables in the data into homogeneous groups. In this study, the classification is carried out using both linear and quadratic algorithms from the discriminant analysis. Linear discriminant analysis from these classifiers determines the group elements and calculates the probability that each element belongs to different groups. Then, the element is assigned to the group with the highest probability score. Linear discriminant analysis assumes that the predictors are normally distributed (Gaussian distribution). It also creates a linear discrimination function that assumes different classes have class-specific elements and equal variance/covariance. Unlike the linear discriminant analysis algorithm, in the quadratic discriminant analysis algorithm, variance/covariance equality is not accepted. The covariance matrix for quadratic discriminant analysis may be different for each class category. Hence, it configures the discriminant function to be quadratic (Hart et al., 2000; Lotte et al., 2018).

#### 3.4.3. Naive Bayes

Naive Bayes is a classifier algorithm that utilizes Bayes' theorem based on probability which is connected to the relationship between marginal and conditional probabilities (Hart et al., 2000). In the working principle of the algorithm, all features are regarded to be independent, and this is also the reason for using the name “Naive”. However, all features have the same effect value on classification, which means each of the features has an equal weight in the training (Tzallas et al., 2009). It is the mostly preferred algorithm in machine learning approaches due to its simple calculation mechanism created by the non-realistic approach (Cura and Akan, 2021; Sayilgan et al., 2021a; Degirmenci et al., 2022b). The Gaussian and Kernel algorithms of this Naive Bayes classifier were computed for this study.

#### 3.4.4. Support vector machine

Support vector machine is a well-known supervised learning algorithm, which is a non-probabilistic approach that uses the geometric characteristics of input data. It is mostly used in both classification and regression studies. *N* dimensional space is created utilizing the elements of the coordinate systems. These elements consist of the data including *n* features. The decision boundaries, which are named “hyperplane”, are generated to discriminate the input data into different classes. Although many hyperplanes can be defined to categorize the different classes in the process, the optimum hyperplane that separates the different classes best is selected to provide a more accurate classification. The distance between the “support vectors” that belong to different class categories is defined as the “margin”. In this algorithm, the maximum margin is a critical parameter. The data placed on different parts of the hyperplane are indicated as a component of a different class (Vapnik, 1999; Hart et al., 2000; Lotte et al., 2018). All different types of support vector machine classifiers were computed in this study, i.e., linear, quadratic, cubic, fine Gaussian, medium Gaussian, and coarse Gaussian algorithms.

#### 3.4.5. K-nearest neighbors (KNN)

KNN is a successful machine learning algorithm that is mostly preferred in classification and regression processes. The learning process is carried out based on the data in this algorithm (Isler et al., 2023). As a first step, the distance between the sample to be predicted and all input data in the training set is calculated. Among the k-nearest neighbors, those which provide the minimum distance are determined. Then, the class of the new sample is indicated as the most common class among these k-Nearest Neighbors (Isler, 2009; Tzallas et al., 2009). The distance calculation can be performed using different distance measurement methods such as Euclidean, Manhattan, Minkowski, and Hamming (Hart et al., 2000). In this study, fine, medium, coarse, cubic, cosine, and weighted algorithms of the k-NN classifier were executed. “Euclidean” distance measurement method is one of the most selected distance calculation methods (Isler, 2009; Cura and Akan, 2021). Hence, it was selected and adopted for the execution of fine, medium, coarse, and weighted algorithms in this study. Additionally, “cubic” and “cosine” distance metrics were used in cubic and cosine algorithms, respectively.

#### 3.4.6. Logistic regression

The basic concept of logistic regression is the modeling of the probability of an event. The probability value is defined as a continuous variable, and two different outputs are available in logistic regression-based classifications. Hence, this algorithm can be used for binary classification studies. In the process, the logistic function which is also defined as the sigmoid function is fitted to the input data utilizing probability (Tzallas et al., 2009). The logistic regression algorithm projects the data points based on a line and all log-odd values evaluated. These log-odd values which are considered inputs are converted to probability values. These calculated probability values are defined as outputs of the algorithm. Hence, the sigmoid function is fitted using this input–output transformation. The different line rotations are tested by calculating, logging, and summing conditional probabilities for all steps. Then, the best fitting function which obtained the maximum probability is evaluated (Alkan et al., 2005).

#### 3.4.7. Ensemble learning

Ensemble learning is a meta-algorithm that combines multiple machine learning techniques into a single prediction model (classifier) to reduce variance (bagging), bias (boosting), and/or improve predictions by preventing the overfitting problem. This algorithm generally assumes that a single classifier cannot achieve certain and precise classification accuracy due to possible noise, overlapping data distributions, and outliers in the data. Hence, this algorithm supposes that there is no single model (classifier) that works best for every classification problem (Sayilgan et al., 2021b). Consequently, recently, ensemble learning methods have become frequently preferred classification algorithms in the recent literature. In this study, the algorithms of Boosted, Bagged, Subspace Discriminant, Subspace k-NN, and RUSBoosted Trees which are developed under ensemble learning classifiers are tested since they have been implemented in Matlab already.

### 3.5. Performance evaluation metrics

In the performance evaluation of classification results, the reel label of MI EEG segments was compared with the predicted label assigned by classifier algorithms. The MI tasks classification results of the classifiers are calculated using true positive (TP), true negative (TN), false positive (FP), and false negative (FN). These values are calculated from the confusion matrix, and they are used to calculate accuracy (ACC) performance metric.

On the other hand, the k-fold cross-validation (CV) method is computed to evaluate classifier performance. k-fold CV randomly separated the extracted feature set as k different folds with equal sizes. Among these folds, the (k-1) fold is used as training data, and the remaining one-fold is used as test/validation data. In each classification, this process is repeated k times, and accuracy values are calculated for each iteration. At the end of the k iterations, the average accuracy value of the classification is calculated. In this study, the k value is chosen as 5 to apply the k-fold CV method. Additionally, 10 repeated tests were performed to check the repeatability of classification results. The mathematical formulas of performance metric computed to evaluate the classifier performance are expressed in the following equations (Hart et al., 2000; Isler, 2009; Degirmenci et al., 2021):


(12) $$\begin{array}{l} ACC\left(\%\right)=\frac{TP+TN}{TP+TN+FP+FN}\times 100 \end{array}$$

Here, while the number of data that actually belongs to a class and is marked to the same class by the classifier is expressed as TP, the number of data incorrectly marked to a different class is also expressed as FN. However, the number of data that actually belongs to a different class and is marked to a different class by the classifier is expressed as TN, and the number of data incorrectly marked to the same class is expressed as FP.

## 4. Results

In this study, we aim to classify MI tasks of EEG segments using all extracted features and statistically significance-based selected features only. As the implementation details of this study, the segmentation of EEG signals, feature extraction, and classification steps in the study was performed in MATLAB application. In the feature selection process, the software package “IBM SPSS Statistics 25”, which is generally used in statistical analysis, was used to perform the independent *t*-test for the 2-class task and the ANOVA test for the multiple-class task. The *p*-values which define the statistical significance are also found using this software program.

EEG signals are supplied from BCI Competition IV Dataset IIa in this study. MI EEG segments are extracted for 22-channel EEG recordings of 9 subjects. The feature set is calculated from temporal, spectral, time-frequency, and non-linear methods. In the time domain, 24 different features were extracted from 22 EEG channels for each MI EEG task sample. Hence, a total of 528 temporal features were supplied for each sample. The detailed description of 528 temporal features is “(number of EEG channels) × (number of features)”. The spectral features, energy, entropy, and variance of EEG sub-frequency bands (*δ*, *θ*, *α*, *β*, and *γ*) were calculated using fast Fourier transform-based frequency band extraction. These spectral features were extracted from 22 EEG channels for each MI EEG task sample. Hence, a total of 330 spectral features were supplied for each sample. In the time-frequency domain, energy, entropy, and variance of EEG sub-frequency bands (*δ*, *θ*, *α*, *β*, and *γ*) were calculated using Wavelet Transform-based frequency band extraction. These time-frequency features were calculated from 22 EEG channels for each MI EEG task sample. Then, a total of 330 spectral features were supplied for each sample. The detailed description for both 330 spectral and 330 time-frequency features is “(number of EEG channels) × (number of frequency subbands) × (number of features)”. As non-linear features, the values of (*SD*_1_) and (*SD*_2_), the product (*SD*_1_x*SD*_2_), and the ratio (*SD*_1_/*SD*_2_) were calculated from 22 EEG channels for each MI EEG task sample. The non-linear features were calculated for 2 different lag conditions, and a total of 176 non-linear features were supplied for each sample in the assumption of both *lag=1* and *lag=9*. The detailed description for 176 non-linear features is “(number of lag conditions) × (number of EEG channels) × (number of features)”. The “(2592 × 1364)” feature vector which includes 2,592 samples and 1,364 features was supplied for multi-task classification at the end of the feature extraction process for all subjects. The “(1296 × 1364)” feature vector which includes 1,296 samples and 1,364 features was supplied for binary classification at the end of the feature extraction process for all subjects. In addition to the feature extraction process, we also aimed to investigate the effectiveness of the statistical significance-based feature selection method for both multi-task and binary classification. The statistically significant features were defined based on the statistical significance level using the ANOVA test and independent *t*-test for multi-task and binary classification processes, respectively. The results of ANOVA-based statistical analysis show that 673 out of 1,364 features yielded a significant *p*-value for multi-task classification. The independent *t*-test-based statistical analysis showed that 91 out of 1,364 features yielded a significant *p*-value for binary classification. The extracted and selected feature sets were given to the classifier algorithms using five-fold cross-validation to predict the MI tasks of samples. Finally, various classifiers such as decision tree, discriminant analysis, Naive Bayes, support vector machine, k-NN, logistic regression, and ensemble learning were utilized for the classification. The results of each classifier algorithm were evaluated based on the 10 repeated tests. Then, the average accuracy values of these repeated tests were evaluated for each classification process.

Tables 1, 2 show the accuracy-based performance evaluation results of the study. In the tables, the highest classification result for the related component is indicated with boldface numbers. The performance evaluation of the binary classification is presented in Table 1. In the table, “1st Task” indicates that the classifications were performed using the feature set combining the time-domain, frequency-domain, time-frequency domain features, and non-linear parameters. On the other hand, “2nd Task” denotes that classifications were performed using the selected feature set by a statistically significant (the independent *t*-test) based feature selection method. Among all 1st task classifications, the highest average accuracy value of 57.30% is achieved using the ensemble boosted trees algorithm and all features. In the 1st task, there are N/A results among classification results due to the fact that the prepared feature set does not provide suitable parameters for the structure of the classifier. In addition, the highest average accuracy value of 61.86% is achieved using the ensemble subspace discriminant algorithm and selected features by the independent *t*-test among all 2nd task classifications.

**Table 1:** Binary classification performance of the time-domain, frequency-domain, time-frequency domain, and non-linear features and the effectiveness of the independent *t*-test-based feature selection.

**Classifier algorithms**	**Classifier accuracies (%)**	
	**1st Task**	**2nd Task**
Fine Tree	54.10	56.50
Medium Tree	56.10	55.20
Coarse Tree	56.60	55.90
Linear Discriminant Analysis	N/A	52.10
Quadratic Discriminant	N/A	52.00
Logistic Regression	N/A	51.10
Gaussian Naive Bayes	48.20	57.10
Kernel Naive Bayes	48.50	55.70
Linear Support Vector Machine	N/A	51.40
Quadratic Support Vector Machine	N/A	51.10
Cubic Support Vector Machine	N/A	50.70
Fine Gaussian Support Vector Machine	N/A	49.40
Medium Gaussian Support Vector Machine	N/A	51.20
Coarse Gaussian Support Vector Machine	N/A	51.20
Fine K-Nearest Neighbors	49.80	50.20
Medium K-Nearest Neighbors	49.80	50.30
Coarse K-Nearest Neighbors	49.80	49.80
Cosine K-Nearest Neighbors	49.80	50.40
Cubic K-Nearest Neighbors	49.80	50.70
Weighted K-Nearest Neighbors	49.80	50.80
Ensemble Boosted Trees	**57.30**	58.40
Ensemble Bagged Trees	53.14	55.91
Ensemble Subspace Discriminant	51.94	**61.86**
Ensemble Subspace K-Nearest Neighbors	50.28	50.69
Ensemble RUSBoosted Trees	55.84	56.32

The 1st task indicates that all features are used, and the 2nd task shows that only *t*-test-selected features are used in the classifier inputs. Bold values indicate the maximum classifier performances for the given task.

**Table 2:** Multi-task classification performance of the time-domain, frequency-domain, time-frequency domain, and non-linear features and the effectiveness of the independent *t*-test-based feature selection.

**Classifier algorithms**	**Classifier accuracies (%)**	
	**1st Task**	**2nd Task**
Fine Tree	31.80	32.51
Medium Tree	34.50	34.50
Coarse Tree	33.10	33.61
Linear Discriminant Analysis	N/A	27.31
Quadratic Discriminant	N/A	25.73
Gaussian Naive Bayes	27.90	29.09
Kernel Naive Bayes	27.30	29.43
Linear Support Vector Machine	25.00	27.42
Quadratic Support Vector Machine	25.00	27.09
Cubic Support Vector Machine	25.00	27.00
Fine Gaussian Support Vector Machine	25.00	26.20
Medium Gaussian Support Vector Machine	25.00	26.78
Coarse Gaussian Support Vector Machine	25.00	26.85
Fine K-Nearest Neighbors	24.90	25.65
Medium K-Nearest Neighbors	24.90	25.80
Coarse K-Nearest Neighbors	24.90	25.68
Cosine K-Nearest Neighbors	24.90	26.21
Cubic K-Nearest Neighbors	24.90	25.67
Weighted K-Nearest Neighbors	24.90	26.00
Ensemble Boosted Trees	**35.60**	36.41
Ensemble Bagged Trees	32.83	35.28
Ensemble Subspace Discriminant	27.14	**47.36**
Ensemble Subspace K-Nearest Neighbors	25.28	28.48
Ensemble RUSBoosted Trees	34.92	34.77

The 1st task indicates that all features are used, and the 2nd task shows that only ANOVA-selected features are used in the classifier inputs. Bold values indicate the maximum classifier performances for the given task.

To investigate the effect of the study on the four-task classification task, the feature set is prepared to extract the same features from EEG signals. Additionally, the significant features are determined using the statistical significance (ANOVA test)-based feature selection method, and the selected feature set is obtained. The four-task classification performance results are presented in Table 2. As the previous table, “1st Task” indicates that the classifications are performed using the feature set by combining the time-domain, frequency-domain, time-frequency domain features, and non-linear parameters. On the other hand, the “2nd Task” indicates that the classifications are performed using the selected feature set by statistical significance (ANOVA test)-based feature selection method. The highest classification average accuracy value of 35.60% is obtained using ensemble boosted trees among all 1st task classifications. On the other hand, the highest classification average accuracy value of 47.36% is obtained with the ensemble subspace discriminant algorithm among all 2nd Task classifications.

In addition to accuracy-based performance evaluations, the sensitivity and specificity values were also calculated for only ensemble subspace discriminant algorithm-based classification since it provides the highest average accuracy value for both the binary and four-task classifications. These results are presented in Table 3. SEN and SPE values are calculated as 47.61% and 82.54% for the four-task classification in the 2nd Task, respectively. On the other hand, for the binary MI task classification, 65.28% SEN and 58.49% SPE values are calculated with the ensemble subspace discriminant classifier in the 2nd Task.

**Table 3:** Comparison of various multi-class and binary Motor Imagery task classification studies with the results of the study.

**Study**	**Channels**	**Classes**	**Classifier**	**ACC (%)**
Degirmenci et al. (2022b)	22	4	Linear Discriminant Analysis	44.00
Degirmenci et al. (2022c)	22	2	Ensemble Subspace Discriminant	62.52
Lu et al. (2020)	2	2	Random Forests	68.32
Kato et al. (2020)	21	5	Support Vector Machines	40.60
Sakhavi et al. (2015)	22	4	Convolutional Neural Network	70.60
Garcia-Laencina et al. (2014)	2	5	Linear Discriminant Analysis	77.30
Jusas and Samuvel (2019)	8	4	Support Vector Machines	56.00
Nguyen et al. (2018)	22	4	Fuzzy Logic System	65.00
Lindig-Leon and Bougrain (2015)	26	4	Linear Discriminant Analysis	51.67
Ma et al. (2018)	64	5	Recurrent Neural Networks	68.20
Xu et al. (2019)	3	2	Convolutional Neural Network	74.20
Zhao et al. (2019)	22	2	Convolutional Neural Network	69.00
Lee et al. (2019)	64	4	Linear Discriminant Analysis	58.20
**This study**	22	4	Ensemble Subspace Discriminant	47.36
**This study**	22	2	Ensemble Subspace Discriminant	61.86

ACC is the highest accuracy achieved in the cited paper. In the list, Kato et al. (2020) used the MISCP dataset, Ma et al. (2018) used EEG Movement/Imagery Database (eegmmidb) dataset, Garcia-Laencina et al. (2014) used 5 different BCI-EEG datasets together, Lindig-Leon and Bougrain (2015) and Lee et al. (2019) used their own datasets, Xu et al. (2019) used the BCI Competition IV Dataset-IIb dataset, and other studies used BCI Competition IV Dataset-IIa dataset.

## 5. Discussion

In this study, we introduced a multi-directional handcrafted feature extraction-based approach for the representation and classification of multi-channel MI EEG signals. In the study, temporal features, spectral features, time-frequency features, and non-linear parameters of EEG signals are extracted. In addition, the effect of the statistical significance-based feature selection method is investigated to indicate significant and effective features from extracted feature set which includes the combination of various MI EEG features. The binary and multi-task classification studies were performed with the same feature extraction approach. In these studies, two different scenarios are available. “1st Task” denotes the classifications of the feature set that includes all features. “2nd Task” denotes the classifications of the selected feature set that includes statistically significant features. The extracted and selected feature sets are classified using 24 different classifier algorithms. In the binary classification process, logistic regression is also used. The accuracy, sensitivity, and specificity-based performance evaluations are performed to analyze the classifier performances implemented in this study.

In the binary classification task, classification could not be performed in 9 of all classifiers, and the highest average accuracy among the remaining classifications was achieved with ensemble boosted trees for the 1st task. On the other hand, for the 2nd task, it was possible to classify with all classifiers and the highest average accuracy value was obtained with the ensemble subspace discriminant algorithm. Moreover, the aim of the study included the investigation of statical significance-based feature selection in binary classification. To investigate the effectiveness of the independent *t*-test-based feature selection approach, 1st and 2nd scenarios of Table 1 are compared. It was observed that the feature selection method based on the independent *t*-test increased the performance in 13 classifiers, decreased the performance in 2 classifiers, and did not change the performance in 1 of them. We should note that considering the significant improvement in classifier performances, the independent *t*-test-based feature selection may be used as an effective feature extraction approach for binary classification studies.

In multi-task classification approaches for the 1st task, two algorithms from all classifiers could not be used for the classification of MI tasks, and the ensemble boosted trees algorithm yielded the highest average accuracy value among the remaining classifiers. In the 2nd Task, all classifiers were used for classification, and the highest average accuracy value was acquired with the ensemble subspace discriminant algorithms. ANOVA test-based feature selection process was performed to predict statistically significant features. Hence, the 1st and 2nd tasks of Table 2 are compared to determine the effectiveness of this statistical significance-based feature selection process on multiple MI task classification. According to Table 2, the classification was performed using ANOVA test selected features for two classifiers. It was observed that the feature selection method based on the ANOVA test increased the performance in 20 classifiers, decreased the performance in 1 classifier, and did not change the performance in 1 of them. Hence, the ANOVA test-based dimensionality reduction of EEG features approach is an effective feature selection method that provides a significant improvement in classifier performances for multiple MI task classification studies. In binary and multiple MI task classification, experimental results revealed that the selected statistically significant features introduced in this study outperform the results achieved using all EEG features.

In Table 3, we summarize some of the previous binary and multiple MI task classification studies and compare their performances with the performance of the study. The details of the studies including dataset, channel selection, feature extraction approaches, feature selection method, classes (binary or multiple), classifier algorithms, and classification performances based on the various metrics (ACC, SEN, and SPE) are given in Table 3 for effective comparison of these studies. In the study by Degirmenci et al. (2022b), the multiple (left hand, right hand, feet, and tongue) tasks were tried to be differentiated using 24 different time-domain features which were extracted from 22 channel EEG signals. On the other hand, the effectiveness of ANOVA-based feature selection was investigated, and the highest average accuracy was calculated as 44.30% using only statistically significant features. Each introduced feature extraction method of EEG signals is a factor that plays a significant role in the classification success of the study. In our study, the frequency domain, time-frequency domain, and non-linear parameters are also introduced, and these features provide higher success rates than that of their study. In the study by Degirmenci et al. (2022c), the independent *t*-test-based feature selection approach was performed using time-domain and frequency-domain features. All EEG channels of the BCI Competition IV Dataset-IIa were used for binary classification, and the highest average accuracy value of 62.52% was obtained. Their results revealed that the independent *t*-test-based feature selection process of that study generally improves the classifier performances. They reported higher average accuracy values than the ones in our study, but in their study, they did not use the time-frequency domain and non-linear features. In another study, Lu et al. (2020) used BCI Competition IV Dataset-IIa, and Wavelet packet decomposition-based binary classification was adopted. The accuracy value of 68.32% was achieved with the random forest classifier algorithm, and the reported value is higher than our binary classification results. However, in that study, both channel selection (C3 and C4) and DFSS-based feature selection processes were conducted. Since the feature selection provides the important features among all features from all EEG channels, a channel selection process is not adopted in our study. In the study by Kato et al. (2020), five finger movements are predicted using 21 EEG channels of the MISCP dataset. Multi-class common spatial pattern-based features were differentiated using support vector machine, and an accuracy value of 40.60% was achieved. Although more EEG channels were evaluated, the reported accuracy value was lower. If they adopted a feature selection algorithm for their study, they might reach a higher classifier performance. In addition, as the number of classes to be classified increases, the success of multi-task classifications remains at lower levels compared to the binary classification as in that study and our study. In another study, Sakhavi et al. (2015), filter-bank common spatial patterns and energy-based features are extracted using 22 EEG channels of BCI Competition IV Dataset-IIa. Then, convolutional neural networks were used to classify four different MI tasks, and an accuracy value of 70.60% was achieved. The reported classification result was higher than the accuracy achieved in our study. Although convolutional neural network-based approaches might increase the classification success, the training time generates a high computational load for the designed system. However, the computational complexity of our feature extraction, feature selection, and classification processes is lower than in convolutional neural network-based studies. In the study by Garcia-Laencina et al. (2014), a feature extraction process including band power features, Hjorth parameters, and adaptive auto-regressive coefficients is presented using five BCI-EEG datasets. Local Fisher discriminant analysis is applied for feature selection. Five-finger movements are classified using the linear discriminant analysis algorithm with an accuracy of 77.30%. The reported accuracy value is higher than the accuracy in our study, but the channel reduction process is conducted in addition to the feature selection, and only C3 and C4 channels are evaluated for their proposed methods. In another study by Jusas and Samuvel (2019), the channel reduction and feature selection processes were conducted, and the authors performed an analysis with 8 EEG channels by applying channel selection and also used the PCA-based feature selection process. PCA-based selected fast Fourier transform and channel variance features of EEG signals are classified with an accuracy of 56.00% using the least squares support vector machine. In the study by Nguyen et al. (2018), common spatial patterns and in the study by Lindig-Leon and Bougrain (2015) common spatial patterns and band power feature extraction methods were applied to classify multiple tasks, achieving higher classification performances than the accuracy in our study. Although more EEG channels are evaluated, only the spatial features are considered, and time domain features, time-frequency domain features, and non-linear features were not included in their feature extraction process. Although common spatial pattern-based feature selection was applied, which is known to have a positive effect on MI task classification performance success, the performance is still not at very high levels for these studies. In another study by Ma et al. (2018), the sliding window method and transposed matrix were used to represent 64-channel EEG signals. They used EEG signals from Movement/Imagery Database (eegmmidb) for the prediction of five classes, and these classes were eye closed (baseline), and tasks imagining moving both feet, both fists, left fist, and right fist. The accuracy value of 68.20% is yielded with recurrent neural networks. The classification accuracy is higher than the accuracy achieved in our multi-task classification task. On the other hand, such deep learning approaches have more computational complexity than our study since they combine feature selection and classification processes. In a binary task classification study by Xu et al. (2019), the time-frequency representations of EEG signals are obtained using the short-time Fourier transform method, and 2D EEG images are given to convolutional neural network structure for classification. The accuracy value of 74.20% is calculated by their proposed approach. Although the success of the study appears to be higher than our study, the computational complexity due to image transformation of EEG signals and convolutional neural network-based classification should not be ignored. In addition to computational load, less number of channels (C3, Cz, and C4) are only evaluated. Considering the high performance of deep learning approaches and the effectiveness of channel reduction on performance, better classification results could be achieved. In another binary classification study by Zhao et al. (2019), Wavelet Transform and convolutional neural network-based approach using 22 EEG channels of BCI Competition IV Dataset-IIa are introduced. The classification result showed that the accuracy value is calculated as 69.00%. As in the previous study, there is no significant performance improvement considering the advantages and drawbacks of convolutional neural network-based approaches, despite the occurred additional computational complexity. In the study by Lee et al. (2019), time-domain parameters are extracted from 64 EEG channels, and a private dataset is used. Four different tasks which are Grasp, Spread, Pronation, and Supination are differentiated with an accuracy of 58.20% using the shrinkage-regularized linear discriminant analysis algorithm. They used more EEG channels and different multiple-task categories, but only temporal features were extracted as EEG features, and the other feature extraction categories were ignored.

Considering the contributions, benefits, and drawbacks of these binary and multiple-task classification studies, some parameters play important roles in the MI task classification process. These are dataset, number of channels, channel selection, feature extraction methods, feature selection methods, classifier algorithms, and number of classes. The main drawback is computational complexity due to feature extraction methods and the classification process of EEG signals. In EEG signal processing, the basic goal is to achieve high-performance values using all channels of EEG signals. Another important aspect is adopting an effective feature selection method that indicates the relevant and discriminative MI EEG features and improves the classifiers' performance. The statistically significant feature-based approach we used in the study, which has computational advantages, resulted in an accuracy of 61.86 and 47.36% for binary classification and four-task classification, respectively. In addition, 22 channels of EEG signals are evaluated for process, and different feature categories which are time-domain, frequency-domain, time-frequency domain, and non-linear are used for feature extraction. The classification results indicate that the statistically significance-based feature selection process is an effective feature selection method that generally improves classifier performances. Therefore, the encouraging performance results of this study with the computational advantages demonstrate that the statistically significant feature-based approach may be applied to other EEG-based studies.

## 6. Conclusion

Decoding of MI tasks has an important role to provide a reliable and convenient way of information interaction for paralyzed patients to control external devices. EEG signals are commonly used in the classification of MI tasks due to ease of recording and low cost. However, the monitoring and analysis of long-term EEG signals are time-consuming and not reliable because of changes in the experiences of experts. Hence, the selection of effective signal processing and classification approaches plays an important role in the accurate analysis of MI EEG signals.

In this study, we extracted features using time-domain, frequency-domain, time-frequency domain features, and non-linear methods. In addition, the effectiveness of the statistically significance-based feature selection method is investigated. The statistically significant MI EEG features are determined using statistical significance (ANOVA test and independent *t*-test)-based feature selection for four tasks and binary task classifications. The results showed that the ensemble learning classifiers (boosted trees and subspace discriminant algorithms) yielded the maximum classifier performance in four tasks and binary task classifications. Ensemble subspace discriminant algorithm yielded accuracy values of 47.36 and 61.86% using the selected feature set including statistically significant MI EEG features for four-task and binary task classifications, respectively. The main contribution of this study is the implementation of Poincare plot measures based on non-linear features to commonly use time-domain, frequency-domain, and time-frequency domain features. In our experiments, we observed that the ANOVA test-based and the independent *t*-test-based feature selection processes provide significant improvements in classifiers' performance. Hence, the statistically significance-based selection is a practical feature selection method and may be used to analyze different EEG signal-based studies. Additionally, this study has the advantage of low computational complexity in terms of feature extraction, feature selection, and classification approaches. Therefore, the statistically significant time-domain, frequency-domain, time-frequency domain features, and non-linear parameters are presented as the novel effective features in this study and successfully implemented to predict binary and multiple MI tasks.

## Data availability statement

Publicly available datasets were analyzed in this study. This data can be found here: https://www.bbci.de/competition/iv/#dataset2a.

## Author contributions

YY, MP, and YI contributed to the conception and design of the study. MD implemented all feature extraction, feature selection, and machine learning algorithms in Matlab. MD and YI wrote the first draft of the manuscript. All authors wrote sections of the manuscript and contributed to the manuscript revision, read, and approved the submitted version.
